# Dr. Evans

**Published:** 1897-12

**Authors:** 


### DR. EVANS.

---

Dr. Thomas W. Evans died suddenly of angina pectoris, in Paris, France, November 14th, 1897, having never recovered from the shock of his wife's death.

Dr. Evans was born in the city of Philadelphia, December 23, 1823, and went to Paris in 1846, where he won a great reputation. Since his advent in the French capital Dr Evans has attended to the teeth of most of the crowned heads of Europe, to say nothing of almost innumerable members of the royal families, excepting Queen Victoria and the Sultan of Turkey. He attended Napoleon and the Empress Eugenie and assisted the latter to escape from Paris in his carriage after the battle of Sedan.

The French Emperor, Napoleon III, was one of Dr. Evans' best friends as well as best patients, and the American dentist, in consequence, was in favor at court. Many of the improvements undertaken by Baron Haussmann, which made Paris the pleasure capital of the world, are said to have been undertaken on the advice of Dr. Evans.

Dr. Evans had confidence in the splendid future of Paris and bought largely of real estate in the direction of the improvements. It was he who helped the Emperor design and carry out the vast improvements around the park of the Bois de Boulogne. The Doctor's investments in the neighborhood of this new fashionable park made him a many-times millionaire. Land bought by him in the early days of the imperial regime for 30 francs the square metre cannot now be obtained for less than 1,000 francs.

Early in the morning of September 4, 1870, the news of the surrender of Marshal McMahon's army, coupled with the abdication and capture of Napoleon III, reached Paris. Mobs began moving on the Tuileries and there was nothing left for the Empress but to escape as quickly as possible from the palace.

The Empress and Mme. Le Breton were thrust by their companions into a cab, but they soon discovered that they only had 3 francs between them, probably not enough to pay the driver, and so they alighted and walked on. Suddenly the Empress realized that they were near the house of Dr Evans and they hurried to him for assistance. Mrs. Evans was out of the city, and so the Empress was given her room and clothing was selected as a disguise. First the doctor tried to get a pass to leave Paris, but failed. He found, however, that one of his friends was in command of the troops at the Bridge of Neuilly, and he succeeded in informing the latter that he would like to pass over the bridge that night with two of his lady patients. The Empress was to play the part of a feeble woman, who was being taken away to a sanatorium. The plan succeeded.

It was Dr. Evans who introduced the Empress to Napoleon. The mother of the Countess of Montijo was first a client and then a friend of Dr. Evans. The mother sent her daughter to Paris as a school girl. The young Spanish beauty as a school girl spent all of her fete days at the house of Dr Evans. It was Dr. Evans himself who took her to a ball given by Napoleon when President. The young lady had heard of the ball and was anxious to go. Her mother had no invitation. as she was in moderate circumstances and was unknown in the Paris world of fashion. Dr. Evans went to the President himself and asked for an invitation for his fair protege. It was at this ball that she was formally presented to Napoleon by Dr. Evans.

Dr. Evans was a very wealthy man, his fortune being estimated at from $25,000,000 to $35,000.000. He visited the United States in August last, bringing with him for interment at Philadelphia the body of his wife. They had no children. While here Dr. Evans is said to have made arrangements to endow schools of dentistry in several of the American universities. The doctor is said to have left large sums of money to charity, and during the course of his life he received innumerable orders and other decorations from foreign potentates.

Among his nephews are Dr. John D'Oyley Evans, of Paris, France, and Dr. W. W. Evans, of Washington City.

Dr. Evans said to an interviewer recently :

"The greatest sorrow I have known was to find my chosen profession deep in the mire. The greatest joy of my life, my proudest achievement and most satisfied thought, is that I have lifted it up from the mud and placed it on the pedestal where it belongs. Year after year I worked, and my best reward is to know that dentistry is now recognized and honored as a profession."

The secret of the success of Dr. Evans as a dentist may be said to be in the fact that he was among the first American dentists to locate in Paris, and also among the first to introduce gold filling for teeth into Europe.

Dr. Evans remained the friend of the United States during the civil war, buying American securities when they dropped to very low points, thus backing up his statements that the Confederacy could not win. The doctor's patriotism had its reward, for the securities eventually built up a large part of his fortune.

Dr. Evans practiced dentistry for a while in Maryland and in Lancaster, Pa., before going to Paris.

